# Nasal nitric oxide in unilateral sinus disease

**DOI:** 10.1371/journal.pone.0171965

**Published:** 2017-02-15

**Authors:** Chia-Hsiang Fu, Hsiao-Jung Tseng, Chi-Che Huang, Po-Hung Chang, Yi-Wei Chen, Ta-Jen Lee

**Affiliations:** 1 Department of Otolaryngology, Head and Neck Surgery, Chang Gung Memorial Hospital and Chang Gung University, Taoyuan, Taiwan; 2 Graduate Institute of Clinical Medical Sciences, College of Medicine, Chang Gung University, Taoyuan, Taiwan; 3 Center for Big Data Analytics and Statistics, Chang Gung Memorial Hospital, Taoyuan, Taiwan; University of South Australia, AUSTRALIA

## Abstract

Unilateral sinus disease (USD) can sometimes be difficult to accurately diagnose before surgery. The application of nasal nitric oxide (nNO) for USD diagnosis and its surgical outcome in USD has not been reported in the literature. We prospectively enrolled sixty-six USD patients who underwent endoscopic sinus surgery for fungal rhinosinusitis (n = 19), chronic rhinosinusitis (CRS) without nasal polyps (n = 13), CRS with nasal polyps (n = 12) and sinonasal mass lesions (n = 22). nNO levels were measured preoperatively and at three and six months postoperatively. Correlations between nNO levels and potential clinical parameters, type of disease, disease severity, and disease-related quality of life (QOL) were assessed. Unlike bilateral CRS, in USD, nNO levels did not correlate with disease severity or postoperative QOL improvements. Except for fungus group, there were no differences in nNO levels between lesion and non-lesion sides in all the other groups. nNO levels on both sides were significantly elevated six months postoperatively in all groups. Fungal rhinosinusitis patients had the lowest preoperative nNO levels, and a cutoff of 239.3 ppb had the best sensitivity (79.0%) and specificity (87.2%) for preoperative diagnosis. While preoperative nNO levels cannot serve as an alternative marker for disease severity of USD, they were lower in fungal rhinosinusitis patients than in other USD patients and may be useful for more accurate diagnosis prior to surgery.

## Introduction

Unilateral sinus disease (USD) is not a rare condition among paranasal sinus diseases encountered in daily practice. Common presentations include unilateral nasal obstruction, purulent nasal discharge, nasal bleeding and facial swelling/painful fullness. Specific diagnosis among the different pathologies of USD made solely by endoscopy or computed tomography (CT) scan can be very difficult, and neoplasms should be considered even if CT scans shows no bony destruction [1]. Although biopsies are frequently necessary before initiating a complete surgical plan [2], a recent review suggested that routine in-office biopsies would not affect clinical management [3].

Nasal nitric oxide (nNO), synthesized from L-arginine by inducible NO synthase (iNOS), is mainly produced in the upper airway, particularly in the paranasal sinus mucosa. Nitric oxide (NO) has been used to monitor local immunity and inflammation, and it contributes to non-specific local host defences against airway pathogens [4]. nNO levels are thought to be affected by rhinosinusitis status and have been implicated in the modulation of cilia beating [5–6]. Thus, nNO has been used to screen for primary ciliary dyskinesia [7] and as a potential postoperative biomarker after sinus surgery for chronic rhinosinusitis (CRS) because nNO levels are well correlated with radiographic staging [8] and symptom severity [9]. In our previous investigation of bilateral CRS with and without polyps, nNO levels were significantly inversely correlated with endoscopy scores and CT scores, elevated significantly after surgery and related to postoperative quality of life (QOL) improvements in both groups [10].

Nasal NO levels can be measured quickly, easily, and non-invasively, and measurements are feasible for following treatment outcomes of bilateral CRS. However, to our knowledge, nNO levels in USD have never been reported. More accurate diagnosis and predicting surgical outcomes preoperatively would benefit USD patients. Regardless of their disease severity, patients are concerned about their prognosis. Thus, the study aimed to assess the merits of nNO for an accurate preoperative diagnosis of USD in patients without bony destruction on CT scans and to determine correlations between nNO levels, surgical outcomes and QOL changes.

## Materials and methods

### Study group

We prospectively recruited patients who underwent unilateral endoscopic sinus surgery (ESS) for USD and were refractory to medication therapy for more than 12 weeks unless a solitary tumor mass was confirmed by endoscopy and biopsy. All study patients were recruited from the Department of Otolaryngology-Head and Neck Surgery and followed for six months or more postoperatively. All patients had paranasal sinus CT scans assessed according to the Lund–Mackay scoring system and met the criteria of the European Position Paper on Rhinosinusitis and Nasal Polyps [11]. We defined the operation side as the lesion side and the contralateral side as the non-lesion side. Patients with the following conditions were excluded: < 18 years of age, prominent contralateral sinus diseases (CT score > 1), obvious bony destruction on CT, previous sinonasal traumas/surgeries/radiotherapies and currently pregnant. Institutional Review Board approval was obtained from Chang Gung Memorial Hospital, Taoyuan, Taiwan. The study was conducted in accordance with the principles of Helsinki Declaration. A written informed consent was obtained from all the subjects.

### Outcome parameters

Patients in this cohort received regular follow-up for approximately six months or more after ESS because QOL is not thought to improve over much longer periods [12]. Subjective evaluation of QOL for patients was performed using the SinoNasal Outcome Test-22 (SNOT-22) on the day before surgery and postoperatively at three and six months as the primary outcome assessment. This instrument was administered by an experienced research assistant blinded to the radiographic staging and the diagnosis. Demographic information on patient age, gender, asthma, or current smoking status was recorded as potential medical variables, and preoperative CT scans were evaluated by a senior surgeon in a blinded fashion to record sinusitis severity using the Lund–Mackay system. For each patient, we also recorded Lund–Kennedy endoscopy score, total nasal resistance by rhinomanometry, allergy test results, and a routine peripheral blood test (eosinophil count, total IgE level) before ESS was performed. The final diagnosis was confirmed by pathology and recorded by physicians postoperatively. The endoscopy score was recorded three and six months postoperatively as an objective outcome measurement.

### Nasal nitric oxide measurements

Measurements were made using an electrochemical analyzer (NIOX MINO^®^; Phadia AB/Aerocrine AB, Sweden) following American Thoracic Society/European Respiratory Society recommendations [13], which we also followed in our previous nNO study of bilateral CRS patients [10]. Briefly, an automatic measurement was set to 45 seconds, and the flow rate of aspiration was set to 5 mL/s. The subject was asked to exhale to tidal volume and a filtered mouthpiece was inserted into his/her mouth and a NIOX nasal olive was inserted into one nostril. The subject then gently inhaled orally to total lung capacity through the mouthpiece while nNO levels were continuously measured. After 45 seconds, the olive was removed from the subject’s nostril, and the nNO values were analyzed within two minutes. The other side was measured in the same manner, and postoperative nNO levels were followed at three and six months postoperatively.

### Statistical analyses

Data were presented as mean ± standard error of mean (SEM) or count with percentage. We used Kruskal–Wallis test and Fisher’s exact test to compare four different diagnostic groups. For each group, nNO levels on the lesion and non-lesion side, pre- and postoperative status of nNO levels, endoscopy scores and QOL scores were compared using the Wilcoxon signed-rank test. Correlation and linear regression analysis were performed to study the relationship between the nNO levels and other variables at the time of pre-operation. Furthermore, by means of ROC curve, we could find the best cutoff value of nNO level to predict fungal rhinosinusitis according to Youden’s index. The endoscopy scores, QOL score, and nNO levels were illustrated by repeated analysis of variance (ANOVA) to show the change by time. Statistical analyses were performed using IBM SPSS (version 20.0) (Armonk, NY: IBM Corp) and GraphPad Prism (version 5) (GraphPad Prism Software, Inc., San Diego, CA). All the *p*-values were 2-tailed, whereas *P* < 0.05 was considered statistically significant and *P* < 0.01 indicated a more marked significance.

## Results

### Demographics and preoperative parameters presented similarly in all groups

Sixty-six consecutive USD patients receiving unilateral ESS for 19 fungal rhinosinusitis (mycetoma), 13 chronic rhinosinusitis without nasal polyps (CRSsNP), 12 chronic rhinosinusitis with nasal polyps (CRSwNP), and 22 sinonasal mass lesions, were enrolled. The sinonasal mass group included nine patients with inflammatory polyps without sinusitis, six with antrochoanal polyps, and seven with inverted papillomas. We excluded all sinonasal malignancies because adjuvant therapy may have interfered with nNO levels. Demographic data and clinical factors are shown in Table 1. There were no significant differences in the basic background parameters, including age, smoking status, allergy status and preoperative QOL scores, between the groups. Potentially related medical variables were analyzed, and endoscopy scores (*P* = 0.008) and CT scores (*P* = 0.002) were significantly higher on the lesion side in the CRSwNP group than in the other groups, as generally expected. The three other groups had similar CT scores.

**Table 1:** Demographic data and clinical parameters.

	Fungus	Mass	CRSsNP	CRSwNP	*P* value
Factors	n = 19	n = 22	n = 13	n = 12	
Age (years)	54.1 ± 1.9	46.8 ± 2.8	52.6 ± 3.4	45.9 ± 3.1	0.060
Gender (male: female)	7: 12	13: 9	7: 6	6: 6	0.548
AR, n (%)	4 (21.0)	11 (50)	4 (30.7)	5 (41.7)	0.260
Asthma, n (%)	1 (5.2)	2 (9.1)	0 (0)	0 (0)	0.520
Smoker, n (%)	2 (10.5)	6 (27.2)	4 (30.7)	4 (33.3)	0.408
CT score	6.8 ± 0.6	5.9 ± 0.6	6.5 ± 0.6	9.8 ± 0.6	0.008*
Endoscopy score	3.4 ± 0.3	4.3 ± 0.4	3.4 ± 0.5	5.1 ± 0.3	0.002*
Nasal resistance (Pa)	0.69 ± 0.12	1.18 ± 0.25	0.87 ± 0.21	0.56± 0.04	0.217
Total IgE (kU/L)	84.3 ± 23.8	87.3 ± 25.5	110.7 ± 52.3	159.0 ± 56.6	0.919
Eosinophil count (%)	1.4 ± 0.3	2.6 ± 0.5	2.4 ± 0.8	2.9 ±1.0	0.252
SNOT-22 score	37.9 ± 5.2	36.1 ± 3.8	37.6 ± 4.2	39.3 ± 5.7	0.977

AR, Allergic rhinitis; CT, computed tomography; n, number of patients; CRSsNP, chronic rhinosinusitis without nasal polyps; CRSwNP, chronic rhinosinusitis with nasal polyps; SNOT-22, SinoNasal Outcome Test-22.

*P level was statistically significant by Kruskal–Wallis test.

### Fungal rhinosinusitis exerted the lower preoperative nNO levels which unrelated to disease severity

We first compared preoperative nNO levels between the lesion and non-lesion sides in each group. In our previous study, we demonstrated that preoperative nNO levels are inversely correlated with CT scores in bilateral CRS patients (CRSsNP and CRSwNP), which indicates that more advanced sinusitis status had lower nNO levels [10]. In this USD cohort, however, the lesion side had a significantly higher endoscopy / CT score, demonstrating nNO levels similar to those on the non-lesion side in all but the fungus group (Table 2). nNO levels on the lesion side were significantly lower than those of the non-lesion side in fungal rhinosinusitis (*P* = 0.028). However, in the fungus group, the correlation between nNO levels and CT scores did not reach a significant level (*P* = 0.258). Indeed, unlike bilateral CRS patients, nNO level did not correlate with disease severity (endoscopy score and CT scores, *P* = 0.074 and 0.486, respectively) or SNOT-22 scores (*P* = 0.159) in this USD cohort.

**Table 2:** Levels of preoperative nasal nitric oxide in every group.

	Lesion side	Non-lesion side	*P* value
Fungus	151.3 ± 18.1	228.5 ± 26.9	0.013*
Mass	365.3 ± 35.5	410.0 ± 43.2	0.270
CRSsNP	400.5 ± 59.4	394.5 ± 58.2	0.893
CRSwNP	404.4 ± 45.2	446.2 ± 54.3	0.530

CRSsNP, chronic rhinosinusitis without nasal polyps; CRSwNP, chronic rhinosinusitis with nasal polyps.

*P level was statistically significant by Wilcoxon signed-rank test.

**Fig 1:**
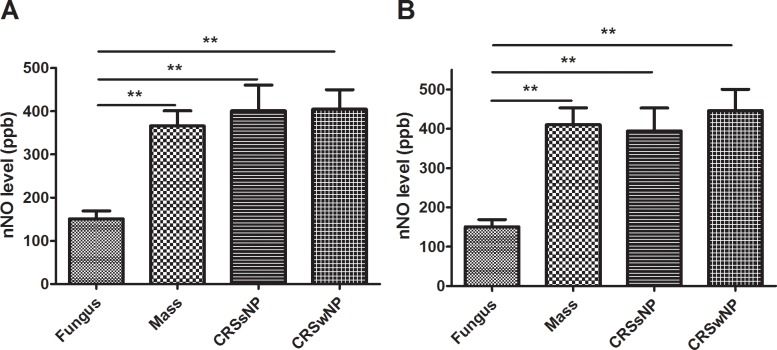
The differences of preoperative nasal nitric oxide (nNO) levels between study groups. Comparison of preoperative nNO on the (A) lesion side and (B) non-lesion side among the groups was analyzed. Data were expressed as the mean ± standard error of mean (SEM). CRSsNP = chronic rhinosinusitis without nasal polyps; CRSwNP = chronic rhinosinusitis with nasal polyps. ^*^P < 0.05, ^**^P < 0.01, Mann–Whitney U test.

The preoperative nNO level was significantly lower on the lesion side (Fig 1A) and non-lesion side in the fungus group (Fig 1B) than in the other groups (by Kruskal–Wallis test, *P* < 0.001 and 0.002, respectively). The other three groups had similar and higher preoperative nNO levels than those of the fungus group, but the latter did not present a more advanced CT score, which indicates that the nNO level in USD could not reflect the status of sinusitis.

Finally, we performed univariate analysis to identify medical factors potentially related to mean nNO level (Table 3), and age and the diagnosis were found to involve nNO levels. Multiple linear regression analysis was conducted subsequently to adjust for age and revealed the diagnosis was the only independent factor that significantly correlated with preoperative mean nNO levels (*P* < 0.001).

**Table 3:** Regression analysis for the preoperative nasal nitric oxide level.

	Univariate analysis			Multiple linear regression		
	Estimate (95% CI)		*P* value	Estimate (95% CI)		*P* value
Diagnosis						
Fungus	Baseline			Baseline		
Mass	189.9	(124.2 to 255.6)	<0.001*	177.9	(85.4 to 270.5)	<0.001*
CRSsNP	235.4	(129.8 to 341.0)	<0.001*	204.2	(102.0 to 306.3)	<0.001*
CRSwNP	197.8	(108.1 to 287.5)	<0.001*	215.2	(107.3 to 323.0)	<0.001*
Age	-4.3	(-7.8 to -0.8)	0.018*	-2.5	(-5.7 to 0.8)	0.132
Gender	-24.3	(-108.4 to 59.9)	0.566			
Nasal resistance	-37.1	(-88.0 to 13.8)	0.150			
Endoscopy score	23.5	(-2.3 to 49.2)	0.074			
CT score	-5.2	(-20.0 to 9.6)	0.486			
Eosinophil	11.7	(-5.3 to 28.7)	0.173			
Total IgE	0.0	(-0.3 to 0.3)	0.921			
AR	69.9	(-16.0 to 155.8)	0.109			
Smoking	4.0	(-94.4 to 102.3)	0.936			
Asthma	-6.3	(-208.7 to 196.1)	0.951			
SNOT-22 score	-1.6	(-3.8 to 0.6)	0.159			

CRSsNP, chronic rhinosinusitis without nasal polyps; CRSwNP, chronic rhinosinusitis with nasal polyps; CT, computed tomography; AR, Allergic rhinitis; SNOT-22, SinoNasal Outcome Test-22.

*P level was statistically significant.

### Cutoff value of preoperative nNO in the diagnosis of fungal rhinosinusitis

**Fig 2:**
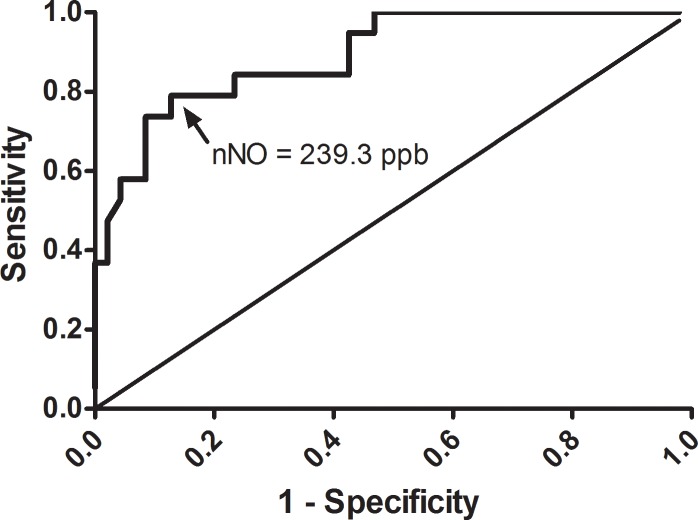
ROC curve for nasal nitric oxide (nNO) in the diagnosis of fungal rhinosinusitis. The cutoff value of nNO level was 239.3 ppb, with a sensitivity of 79.0% and specificity of 87.2% to diagnose a fungal rhinosinusitis.

Sometimes USD is hard to distinguish solely by endoscopy or CT scan preoperatively. However, since in this study fungal rhinosinusitis (mycetoma) had a significantly lower nNO level than those of the other USD groups, we tried to determine a cutoff value by receiver operating characteristic (ROC) curve analysis. According to the Youden’s index, the optimal cutoff of 239.3 ppb nNO to predict fungal rhinosinusitis obtained 79.0% in sensitivity and 87.2% in specificity (area under the ROC curve = 0.892; Fig 2), and the estimated odds ratio was 25.6 (95% CI, 6.34–103.56; *P* < 0.001). The sensitivity was much higher than that for intrasinus calcification on CT which was detected in 11 (57.9%) of the 19 fungus group patients.

We then screened patients by using the preoperative mean nNO level < 239.3 ppb and intrasinus calcification on CT scans. By combining these screening conditions, sensitivity and specificity for the detection of fungal rhinosinusitis in USD increased to 94.7% and 93.3%, respectively. Only 1 fungal and 3 non-fungal rhinosinusitis in this USD cohort would be missed by these screening conditions. We further discovered that in fungal rhinosinusitis patients, those without intrasinus calcification on CT had lower mean nNO levels, but that this difference was not significant (160.9 vs. 210.9, *P* = 0.231).

### Endoscopy scores and QOL improvements not related to pre- and postoperative nNO levels

**Fig 3:**
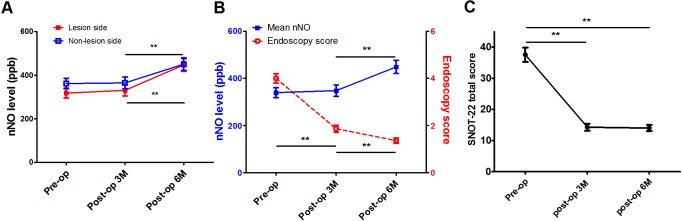
The change of endoscopy scores, QOL score, and nNO levels before and after surgical treatment. Pre- and postoperative status of (A) nasal nitric oxide (nNO) levels on the lesion and non-lesion sides; (B) relationship between endoscopic scores and mean nNO levels; (C) SinoNasal Outcome Test-22 (SNOT-22) total scores. Pre-op = preoperative; Post-op = postoperative; M = months. ^*^P < 0.05, ^**^P < 0.01, repeated ANOVA, with Bonferroni adjustment for multiple comparisons.

All USD patients completed three and six month postoperative evaluations. For the lesion side, nNO levels showed a continuously rising trend but only reached significance six months postoperatively (*P* < 0.001) (Fig 3A). While nNO level on the non-lesion side showed a similar trend by six months postoperatively (*P* = 0.001), endoscopy scores improved gradually from three months after surgery and continued to improve until six months (both *P* < 0.001). Improvements of percentage in endoscopy scores were inversely correlated with elevation of percentage in mean nNO levels postoperatively but not reached significant levels (*P* = 0.116 and 0.292 for three and six months after surgery, respectively) (Fig 3B).

Results show that SNOT-22 scores improved significantly and plateaued from three months until six months postoperatively in all patients (*P* < 0.001) (Fig 3C). Improvements in QOL were observed in each group, which showed that treatment with ESS provided effective symptom relief for all USD patients. However, in contrast to the results in our previous series for bilateral CRS [10], preoperative nNO level for USD patients did not correlate with postoperative improvements of percentage in SNOT-22 scores (*P* = 0.856 and 0.465 for three and six months after surgery, respectively). Accordingly, preoperative nNO levels were not predictors of surgical outcomes for USD patients prior to surgery. Linear regression analysis showed that only preoperative SNOT-22 scores were predictive of improvements in postoperative SNOT-22 scores; indeed, worse preoperative SNOT-22 scores resulted in greater improvements (both *P* < 0.001 for the three and six months postoperative periods), as generally expected.

## Discussion

Most previous studies have reported lower nNO levels in patients with diseased sinus status and that NO level is closely associated with disease severity [5,8–9]. Indeed, in our previous series of bilateral CRS, similar to the results of other studies, nNO levels were inversely correlated with endoscopy scores and CT scores in the CRSsNP and CRSwNP groups [10]. In this study, however, nNO level did not correlate with the endoscopy / CT score in any group of USD patients. Except for the fungus group, nNO level on the lesion side was similar to that on the non-lesion side in all the other groups. A balance effect of nNO was subsequently proposed in USD that NO in lesion side decreased first and followed by the non-lesion side via the communication of nasopharynx, connecting both sides of sinonasal tracts. Thus fungus group had the lowest level of nNO not only in lesion side but also in non-lesion side in patients with USD. In addition, unlike the results of the bilateral CRS study, preoperative nNO level was not correlated with postoperative improvements in endoscopy / SNOT-22 scores. Consequently, nNO level is not useful for assessing preoperative disease severity, or for predicting surgical outcomes, in USD.

In previous study, nNO levels recovered significantly postoperatively in bilateral CRS patients, and the CRSwNP group showed more rapid elevation as soon as three months once the blockage in the sinonasal pathways by nasal polyps was corrected and ventilation of sinuses restored [10]. In this study, however, for patients with USD, nNO levels for the lesion and non-lesion sides both tended to show increases by the third month but reached a significant level at six months after surgery. We speculate that activity of iNOS recovered on the lesion side and had a direct re-ventilation effect after sinus surgery. However, on the non-lesion side, where sinus status was theoretically unchanged because surgery was on the lesion side, the preoperative nNO level was lower than normal [14] and elevated gradually to a significant level concurrent with the increase on the lesion side. Thus, nNO restoration was balanced in the bilateral sinonasal cavities in all groups of USD postoperatively.

Although nNO level was not found to be useful as a prognostic factor for surgical outcomes of USD, it was useful for preoperative detection of fungal rhinosinusitis (mycetoma) more precisely. Intrasinus calcification found on CT images is helpful in preoperative diagnosis but has been detected only in approximately 15–50% of fungal rhinosinusitis patients [15–17]. Because fungal rhinosinusitis in this study was found to have a significantly lower preoperative nNO level, we compared nNO levels with those of other USD patients rather than healthy controls to assess its value as a preoperative diagnostic tool, and a preoperative nNO cutoff level was determined using a ROC curve. We found that preoperative nNO levels lower than 239.3 ppb more accurately identified fungal rhinosinusitis patients than the use of intrasinus calcification on CT before surgery. This decreased nNO level had much better sensitivity and specificity than typical radiologic finding. Accordingly, it could be exploited as a screening aid to make this diagnosis preoperatively and applied in daily practice for patients with USD. A combination of a low preoperative mean nNO level and intrasinus calcification on CT provides even better accuracy for the identification of fungal rhinosinusitis preoperatively, and once sinus aeration has been re-established, good surgical outcomes (a recurrence rate of 6.8%) for fungal rhinosinusitis could be expected [18]. The role of nNO in airway fungal diseases is still unclear. Nitric oxide has been reported to be associated with fungal clearance in hypodermis [19]. On the other hand, fungal pathogen was proved to inhibit the activity of iNOS, resulting in a dose-dependant NO suppression in an animal model [20]. To conclude the association between significantly lower nasal NO (nNO) and fungal rhinosinusitis needs further investigation. Considering the relatively small sample size in this investigation, more USD patient enrolment and longer follow-up would be crucial to make a more definite conclusion.

Collectively, while preoperative nNO levels were not useful for assessing USD severity and prognosis, they were lower in fungal rhinosinusitis patients than in other USD patients and thus may be useful to provide the diagnosis of these patients prior to surgical treatment conducted.

## References

[1] Ikeda K, Tanno N, Suzuki H, Oshima T, Kano S, Takasaka T (1997) Unilateral sinonasal disease without bone destruction. Differential diagnosis using diagnostic imaging and endonasal endoscopic biopsy. Arch Otolaryngol Head Neck Surg 123:198–200. 9046289 10.1001/archotol.1997.01900020082012

[2] Eichel BS (1977) The medical and surgical approach in the management of the unilateral opacified antrum. Laryngoscope 87:737–750. 850452 10.1002/lary.5540870509

[3] Paz Silva M, Pinto JM, Corey JP, Mhoon EE, Baroody FM, Naclerio RM (2015) Diagnostic algorithm for unilateral sinus disease: a 15-year retrospective review. Int Forum Allergy Rhinol 5:590–596. 10.1002/alr.21526 25880633 PMC4830336

[4] Wink DA, Hines HB, Cheng RY, Switzer CH, Flores-Santana W, Vitek MP, et al (2011) Nitric oxide and redox mechanisms in the immune response. J Leukoc Biol 89:873–891. 10.1189/jlb.1010550 21233414 PMC3100761

[5] Ragab SM, Lund VJ, Saleh HA, Scadding G (2006) Nasal nitric oxide in objective evaluation of chronic rhinosinusitis therapy. Allergy 61: 717–724. 10.1111/j.1398-9995.2006.01044.x 16677241

[6] Jain B, Rubinstein I, Robbins RA, Leise KL, Sisson JH (1993) Modulation of airway epithelial cell ciliary beat frequency by nitric oxide. Biochem Biophys Res Commun 191:83–88. 10.1006/bbrc.1993.1187 7680560

[7] Wodehouse T, Kharitonov SA, Mackay IS, Barnes PJ, Wilson R, Cole PJ (2003) Nasal nitric oxide measurements for the screening of primary ciliary dyskinesia. Eur Respir J 21:43–47. 12570107 10.1183/09031936.03.00305503

[8] Deroee AF, Narahhi M, Sontou AF, Ebrahimkhani MR, Dehpour AR (2009) Nitric oxide metabolites as biomarkers for follow-up after chronic rhinosinusitis surgery. Am J Rhinol Allergy 23:159–161. 10.2500/ajra.2009.23.3289 19401041

[9] Jeong JH, Yoo HS, Lee SH, Kim KR, Yoon HJ, Kim SH (2014) Nasal and exhaled nitric oxide in chronic rhinosinusitis with polyps. Am J Rhinol Allergy 28:e11–16. 10.2500/ajra.2014.28.3984 24717870

[10] Fu CH, Huang CC, Chen YW, Chang PH, Lee TJ (2015) Nasal nitric oxide in relation to quality-of-life improvements after endoscopic sinus surgery. Am J Rhinol Allergy 29:e187–191. 10.2500/ajra.2015.29.4249 26637567

[11] Fokkens WJ, Lund VJ, Mullol J, Bachert C, Alobid I, Baroody F, et al (2012) EPOS 2012: European position paper on rhinosinusitis and nasal polyps 2012. A summary for otorhinolaryngologists. Rhinology 50:1–12. 10.4193/Rhino50E2 22469599

[12] Soler ZM, Smith TL (2010) Quality-of-life outcomes after endoscopic sinus surgery: how long is long enough? Otolaryngol Head Neck Surg 143:621–625. 10.1016/j.otohns.2010.07.014 20974329 PMC2965172

[13] ATS/ERS Recommendations for standardized procedures for the online and offline measurement of exhaled lower respiratory nitric oxide and nasal nitric oxide, 2005. Am J Respir Crit Care Med 171,912–930. 10.1164/rccm.200406-710ST 15817806

[14] Phillips PS, Sacks R, Marcells GN, Cohen NA, Harvey RJ (2011) Nasal nitric oxide and sinonasal disease: a systematic review of published evidence. Otolaryngol Head Neck Surg 144:159–169. 21634057 10.1177/0194599810392667

[15] Yoon JH, Na DG, Byun HS, Koh YH, Chung SK, Dong HJ (1999) Calcification in chronic maxillary sinusitis: comparison of CT findings with histopathologic results. AJNR Am J Neuroradio 20:571–574. PMC7056005 10319962

[16] Sidhu R, Mehta N, Dharaviya B, Shah H, Dave A, Chudasama N (2014) Clinico-radiological manifestations of invasive and non-invasive fungal infections in sinuses and respiratory tract. GCSMC J Med Sci 3:42–47.

[17] Ferguson BJ (2000) Fungus balls of the paranasal sinuses. Otolaryngol Clin North Am 33:389–398. 10736412 10.1016/s0030-6665(00)80013-4

[18] Ferreiro JA, Carlson BA, Cody DT 3rd (1997) Paranasal sinus fungus balls. Head Neck 19:481–486. 9278755 10.1002/(sici)1097-0347(199709)19:6<481::aid-hed4>3.0.co;2-v

[19] Li DD, Yang CC, Liu P, Wang Y, Sun Y (2016) Effect of nitric oxide on the antifungal activity of oxidative stress and azoles against candida albicans. Indian J Microbiol 56:214–218. 10.1007/s12088-016-0580-x 27570314 PMC4984430

[20] Collette JR, Zhou H, Lorenz MC (2014) Candida albicans suppresses nitric oxide generation from macrophages via a secreted molecule. PLoS One 9:e96203 10.1371/journal.pone.0096203 24755669 PMC3995984

